# Automated In Situ Seed Variety Identification via Deep Learning: A Case Study in Chickpea

**DOI:** 10.3390/plants10071406

**Published:** 2021-07-09

**Authors:** Amin Taheri-Garavand, Amin Nasiri, Dimitrios Fanourakis, Soodabeh Fatahi, Mahmoud Omid, Nikolaos Nikoloudakis

**Affiliations:** 1 Mechanical Engineering of Biosystems Department, Lorestan University, Khorramabad P.O. Box 465, Iran; 2 Department of Biosystems Engineering and Soil Science, University of Tennessee, Knoxville, TN 37996, USA; anasiri@utk.edu; 3 Laboratory of Quality and Safety of Agricultural Products, Landscape and Environment, Department of Agriculture, School of Agricultural Sciences, Hellenic Mediterranean University, 71004 Heraklion, Greece; dimitrios.fanourakis82@gmail.com; 4 Department of Mechanical Engineering of Biosystems, Urmia University, Urmia P.O. Box 165, Iran; sodabe-fatahi@yahoo.com; 5 Department of Mechanical Engineering of Agricultural Machinery, University of Tehran, Karaj P.O. Box 4111, Iran; omid@ut.ac.ir; 6 Department of Agricultural Sciences, Biotechnology and Food Science, Cyprus University of Technology, Limassol CY-3603, Cyprus; n.nikoloudakis@cut.ac.cy

**Keywords:** *Cicer arietinum*, convolutional neural network, grad-CAM, image classification, ImageNet, VGGNet, VGG16

## Abstract

On-time seed variety recognition is critical to limit qualitative and quantitative yield loss and asynchronous crop production. The conventional method is a subjective and error-prone process, since it relies on human experts and usually requires accredited seed material. This paper presents a convolutional neural network (CNN) framework for automatic identification of chickpea varieties by using seed images in the visible spectrum (400–700 nm). Two low-cost devices were employed for image acquisition. Lighting and imaging (background, focus, angle, and camera-to-sample distance) conditions were variable. The VGG16 architecture was modified by a global average pooling layer, dense layers, a batch normalization layer, and a dropout layer. Distinguishing the intricate visual features of the diverse chickpea varieties and recognizing them according to these features was conceivable by the obtained model. A five-fold cross-validation was performed to evaluate the uncertainty and predictive efficiency of the CNN model. The modified deep learning model was able to recognize different chickpea seed varieties with an average classification accuracy of over 94%. In addition, the proposed vision-based model was very robust in seed variety identification, and independent of image acquisition device, light environment, and imaging settings. This opens the avenue for the extension into novel applications using mobile phones to acquire and process information in situ. The proposed procedure derives possibilities for deployment in the seed industry and mobile applications for fast and robust automated seed identification practices.

## Highlights

A CNN approach for automatic identification of chickpea varieties by seed images.
Two camera types were employed, while light and imaging conditions varied.
The VGG16 architecture was modified by adding four layers.
An average classification accuracy of over 94% was obtained.
Seed industry use possibilities and mobile applications can be derived.

## 1. Introduction

For agriculture, seeds represents the first crucial input. Employing seeds of the appropriate variety is a prerequisite to reach the optimum yield potential and secure a uniform product. Compromised seed varietal purity adversely affects cultivation practices, and eventually limits plant growth and productivity. On this basis, there is a growing pressure on the seed producers, processors, and distributors to assert seed purity. Varietal impurity and misidentification may be introduced in any step of the seed production chain, expanding from cultivation of the mother plants (pre-basic, basic, and certified material) to processing. Varietal impurity may also be the result of intentional adulteration via including seeds of another variety of reduced cost. In this perspective, methods of variety identification and discrimination are highly needed.

Manual seed grading based on morphology is frequently performed by trained experts. This is a time-consuming process, requiring dedicated personnel and variety miss-identification cannot be eliminated. Seed variety identification may be accurately performed by genetic marker-based methods. However, the pitfalls (invasive, time-consuming, and costly) carried by this approach set it unsuitable for routine analysis. In this perspective, there is a growing demand for rapid, non-invasive, and unbiased techniques of variety identification and discrimination.

As non-invasive methods, imaging approaches for seed variety identification and testing include hyperspectral imaging [1,2], near infrared imaging [3,4], and magnetic resonance imagers [5,6]. However, these techniques are characterized by the drawbacks of necessitating costly equipment (conducted by specialized personnel), while subsequent data analysis is highly sophisticated and complicated.

Visible image processing techniques (400–700 nm) offer the advantages of being low-cost and rapid [7,8]. They have been successfully employed across several studies for seed variety identification generally utilizing the most important visual seed features [9]. Rapeseed variety recognition has been conducted by using a method based on computer vision and machine learning [10]. Respective work on rice employed image processing and sparse-representation-based classification methods [11]. Despite being successful and promising, the above-mentioned techniques are based on manual feature engineering in the classification tasks, requiring distinct sections (i.e., feature extraction, selection, and learning). On this basis, there is a necessity for new approaches that can automatically handle these distinct sections. Feature engineering-based approaches additionally require defined illumination conditions during image acquisition. The required specific illumination restricts the applicability of the technique to controlled-light environments [8]. A method independent of the ambient light conditions would be cost-effective, portable, and could be readily employed in the seed industry.

Unlike feature engineering (extraction and selection), where features are created by experts, the feature learning step employs an unattended machine learning algorithm to pick up and create useful features from raw data. The deep learning technology has been increasingly applied for automated tasks (e.g., pattern recognition and image classification), due to its dual ability of automatically extracting efficient visual features directly from input images, and learning complex and vast systems [12,13,14]. Therefore, the potential of deep learning methods is expected to be more powerful and user-friendly than manually engineered features.

Convolutional neural network (CNN) is a specific class of deep learning and a set of non-linear transformation functions. Since 2000, CNN has presented unparalleled success and high performance to object detection, segmentation and recognition, and in this way effectively addressed many classification problems [15]. CNN can automatically perform the feature extraction and selection, owing to the network depth and weight sharing among nodes which assist in reducing over-fitting [16]. CNN has many layers that increasingly calculate image features. Convolutional and fully connected layers are generally two different types of layers on CNN. Input and output in convolutional layers are images that highlight unique patterns, while fully connected layers produce an enhanced vector from prior inputs.

The aim of this study was to develop a proof-of-concept method for variety identification and discrimination by employing CNN. Such models are gradually gaining popularity in deciphering agricultural enigmas such as seeds classification [17,18,19]; however mainly focusing on interspecies variations. Since low-cost image acquisition tools were used, the proposed method can be readily employed in industrial applications, considerably reducing processing time, labor, and associated errors. Chickpea (*Cicer arietinum* L.) was employed as the model species, since previous research on seed variety identification is relatively absent and several diverse types (desi and kabuli) can be used. Chickpea is considered as one of the earliest domesticated legumes, since millennian-old remains have been discovered in the Fertile Crescent and the broader Middle East region [20]. It serves as a nutritional powerhouse (fiber and protein source) to the majority of vegetarians globally, and to individuals where vegetarian attitude is mostly based on economic income in developing countries. In Iran, the area under chickpea cultivation is approximately 450,000 ha, and is mostly (>98%) located in dryland areas (http://www.fao.org/faostat/en/#data/QC accessed on 6 July 2021)), under sustainable cultivation schemes.

## 2. Materials and Methods

### 2.1. Chickpea Seed Samples

**Figure 1:**
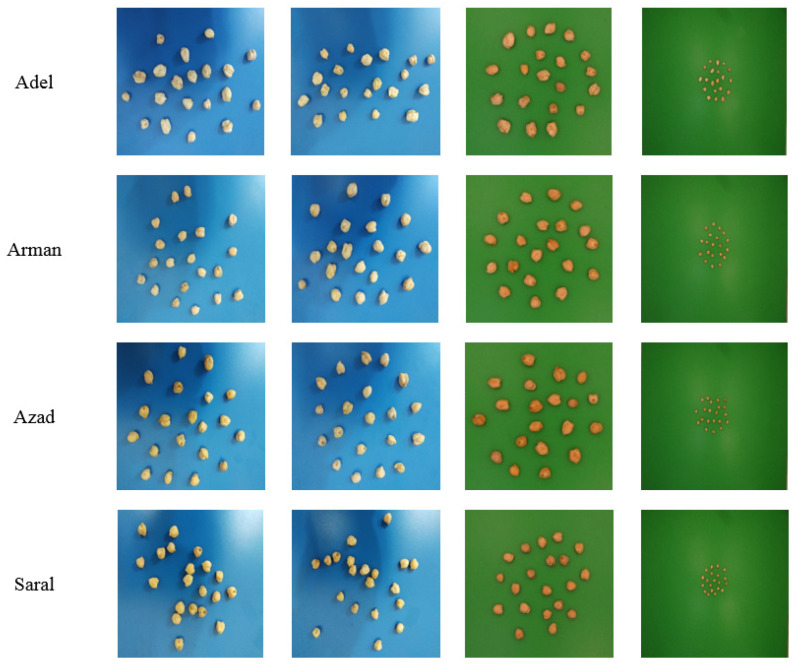
Representative seed images of the four chickpea varieties under study. Twenty seeds randomly placed were used for each picture (1st and 2nd column, randomly zoomed using a blue background; 3rd column, randomly zoomed using a green background; and 4th column, no zoom using a green background); Optical/Digital zoom, source, focus and background were not fixed.

Four commercially prominent chickpea varieties (Adel, Arman, Azad, and Saral) were evaluated. For seed image acquisition, both a mobile phone camera (LG V20, 16-megapixel resolution; LG Electronics Inc., Seoul, Korea) and a digital camera (PowerShot SX260 HS, 12.1-megapixel resolution and 6.2 × 4.6 mm sensor; Canon, Kyoto, Japan) were employed. The latter has a small sensor size, which is comparable with the cameras of mobile phones. During image acquisition (in total 400 images, 100 samples for each variety were obtained), lighting conditions and image capture settings (i.e., background, focus, capturing angle, and camera-to-sample distance) were not fixed. Representative images are displayed in Figure 1.

### 2.2. Deep Convolutional Neural Network-Based Model

CNN consists of convolutional and pooling layers to train red, green, and blue (RGB) images. Convolutional layers involve filters (the so-called kernels) that are used for extracting low-level features (e.g., instance edges and blobs), while pooling layers are applied for the image size reduction [21]. In the CNN structure, common fully connected layers can be employed as in neural networks. The CNN training (according to the comparison between network output and actual labeled output) includes the feed-forward and back-propagation stages. During the feed-forward stage, the image data is applied as the input of the network. In the back-propagation stage, the parameter gradients are obtained using the calculated error in feed-forward, and then each parameter is upgraded according to the calculated gradients [22].

Numerous powerful pre-trained CNN constructions were effectively trained using a great number of labeled images, such as ImageNet which includes 1000 several classes [23]. By employing the obtained chickpea seed variety images, the network-extracted features were not applicable for variety classification. This limitation was resolved by employing the fine-tuning technique. This technique, as a transfer learning concept, was applied to reuse the model. During this procedure, the new data were used to update the model parameters. This indicates that the available network weights were utilized to start the network training procedure instead of initiating with random weights [24].

Commonly employed pre-trained CNNs include AlexNet [25], GoogLeNet [26], ResNet [27], and VGGNet [28]. In the current research, VGGNet was selected due to its superlative performance in the classification operations [24,29]. One of the VGGNet architectures is the VGG-16, which comprises more than 15 million parameters of convolutional layers.

**Figure 2:**
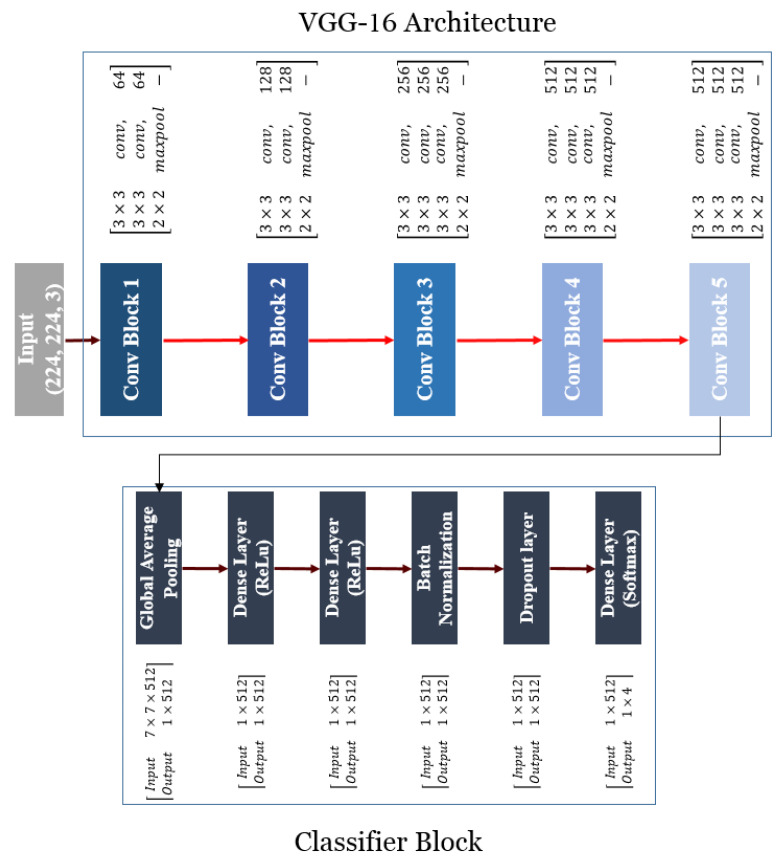
Schematic of VGG-16 architecture (**top panel**) and classifier block structure (**bottom panel**) evaluated in the current research.

Homogeneous construction of VGG-16 involves five blocks in sequence, while the output of each block is specified as the input of the next block (Figure 2). Extraction can exploit the powerful image features, including shape, color, and texture. Two convolutional layers were utilized to build the first two blocks, and three convolutional layers for the last three blocks. The stride and padding of these 13 convolutional layers have 3 by 3 kernels, which are equivalent to 1 pixel, and the Rectified Linear Unit (ReLU) function was applied for each one. A 2 by 2 max-pooling layer with stride of 2 followed each block. This layer was utilized as a sub-sampling one and was employed for dimension reduction of the feature map. Overall, 64 filters were applied to the first block of convolutional layers. In every next block, the number of filters was increased by a factor of 2 as compared to the previous block. At the end, the network had three dense layers.

There were 4096 channels in the first two layers, and 1000 channels in the last layer with a softmax activation function [28]. The last three dense layers were replaced by the classifier block to modify the original VGG-16 architecture.

The global average pooling layer (dense layer) included the ReLU function as the activation function. It also included batch normalization for holding the inputs of layers on the same range. Additionally, it involved dropout as regularization technique for decreasing the overfitting risk of training. The final dense layer with softmax classifier was organized with the classifier block. The last layer had four neurons in order to confirm the network to the present case study.

By minimizing overfitting, the global average pooling layer decreased the number of parameters. In this way, the spatial dimensions of an h × w × d tensor was decreased to 1 × 1 × d dimensions. Each h × w feature map was transformed into a single number according to the mean values of the h × w matrix [30]. The ReLU function was conducted by carrying out the mathematical operation (Equation (1)) on each input data (Parameter *x* in Equation (1)). The Softmax function (Equation (2)) was used to estimate the normalized possibility value of each neuron ($p_{i}$):

(1) $$f\left(x\right)=\{\begin{array}{l} x,\;if\;x>0\\ 0,\;otherwise \end{array}$$

(2) $$p_{i}=\frac{\exp\left(a_{i}\right)}{\sum_{j=1}^{n}\exp\left(a_{j}\right)}$$

Parameter *a_i_* denotes the softmax input for node *i* (class *i*) and i, j ∈ {1, 2, …, n}, where n is the number of classes [31].

### 2.3. Training CNN Model

CNN includes millions of parameters, and its learning requires huge training data and costly calculations. Consequently, feature extraction by a pre-trained network and fine-tuning of the pre-trained network (i.e., transfer learning) are considered as the fundamental strategies to cope with this limitation [15].

For the VGG-16-based model, the fine-tuning process was performed, while training was conducted by the ImageNet dataset [23]. In this way, the information acquired by ImageNet was transferred to the existing case study. At first, VGG-16 was trained by ImageNet. Next, the last fully connected layers were replaced with another classifier block. Training of this block was deployed through random weights for 10 epochs, while the whole convolutional blocks were frozen. Eventually, training of the VGG-16-based model was performed with the dataset of the obtained chickpea seed variety images. Cross-entropy loss function [32] and Adam optimizer [33] were used for procedure training with 0.0001 learning rate and 2e^−6^ learning rate decay.

The VGG-16-based model contained over 15 million trainable parameters. Owing to the great number of parameters, the network overfitting risk was developed. In order to develop the robustness and generalizability of the network, image processing techniques, a slight distortion (comprised of rotation, height and width shifts), and scaling changes were applied to augment the training of a given image.

### 2.4. K-Fold Cross-Validation and Assessment of Classification Accuracy

The VGG16-based model followed by the classifier block was applied for automatic chickpea variety identification and discrimination. The construction of the classifier block was comprised of a global average-pooling layer, various dense layers (1 × 512 neurons and 1 × 512 neurons), a batch normalization layer, and a dropout layer (Figure 2). Training of the CNN network was performed for 100 epochs in every five folds (Figure S1). The weight matrix of the model was acquired according to the minimum loss function value without any over-fitting.

**Figure 3:**
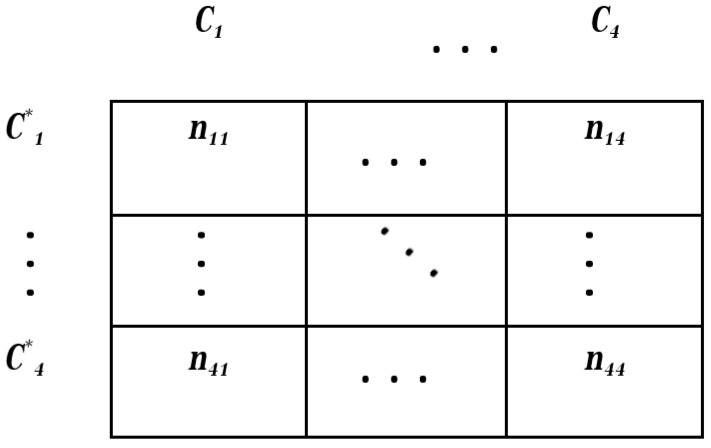
Confusion matrix for classification of four classes.

The collective image dataset was divided into training (80% of images) and test (20% of images) sets. Then, a five-fold cross-validation was performed to evaluate the uncertainty and predictive efficiency of the CNN model. Thus, the training dataset was divided into five disjoint equal segments. Then, four out of five subsets were used as the training sets, while the remaining one as the validation set for training the CNN model. Next, a confusion matrix (Figure 3) was estimated for the assessment of classification efficiency on the independent test data [34].

The terms (*n_ij_*) correspond to the pixels that are classified into class number *i* by the CNN classifier (i.e., C^*^_i_), when they actually belong to class number *j* (i.e., C_j_, *i* = 1, 2, 3, 4). Accordingly, the right diagonal elements (*i* = *j*) correspond to correctly classified instances, while off-diagonal terms (*i* ≠ *j*) represent incorrectly classified ones. When considering one class *i* in particular, one may distinguish the following four instances. True positives (*TP*) and false positives (*FP*) are instances correctly and incorrectly identified as C^*^_i_. True negatives (*TN*) and false negatives (*FN*) are instances correctly and incorrectly rejected as C^*^_i_, respectively. The corresponding counts are determined as $\;n_{TP}=n_{i,i}$,$\;n_{FP}=n_{i,+}-n_{i,i}$, $n_{FN}=n_{+,j}-n_{i,i}$ and $n_{TN}=n-n_{TP}-n_{FP}-n_{FN}\;$ where $n_{i,+}\;and\;n_{+,j}$ are the sums of the confusion matrix elements over row *i* and column *j*, respectively.

The classification performances were measured based on the values of the confusion matrix, and included accuracy (Equation (3)), precision (Equation (4)), sensitivity (Equation (5)), specificity (Equation (6)), and area under the curve (*AUC*; Equation (7)).

(3) $$Accuracy=\frac{n_{TP}+n_{TN}}{n_{TP}+n_{TN}+n_{FP}+n_{FN}}$$

(4) $$Precision=\frac{n_{TP}}{n_{TP}+n_{FP}}$$

(5) $$Sensitivity\left(Recall\right)=\frac{n_{TP}}{n_{TP}+n_{FN}}$$

(6) $$Specificity=\frac{n_{TN}}{n_{TN}+n_{FP}}$$

(7) $$AUC=\frac{1}{2}\left(\frac{n_{TP}}{n_{TP}+n_{FN}}+\frac{n_{TN}}{n_{TN}+n_{FP}}\right)$$

Accuracy focuses on the overall effectiveness of the CNN classifier. Precision evaluates the class agreement of the data labels with the positive labels defined by the classifier. Sensitivity shows the effectiveness of the CNN classifier to recognize positive labels. Specificity denotes how effectively a classifier identifies negative labels. *AUC* indicates the ability of the CNN classifier to avoid false classification.

The confusion matrix values demonstrate the actual and predicted classes. This process was repeated five times through the shifting validation set. Assessment of the CNN classifier performance was done by using the evaluated statistical parameters according to the confusion matrix values in each repetition. These statistical parameters included accuracy, precision, sensitivity, specificity, and AUC. Lastly, overall efficiency of the CNN model was obtained by the average value of the five performances [34,35]. All steps regarding creating and training the proposed CNN model were executed under the Google Collaboratory environment using Python 3.7 and Keras with TensorFlow backend.

## 3. Results and Discussion

### 3.1. Evaluation of Quantitative Classification

For the training dataset, the average values of classification accuracy and cross-entropy loss were 0.9131 and 0.2529, respectively (Table 1). For the validation dataset, the corresponding values were 0.9333 and 0.1902, respectively. For the test dataset, the average values of prediction accuracy and cross-entropy loss were 0.8841 and 0.3943, respectively.

In statistical clustering, algorithms, and models for the classification of data into a determinate set of classes are generally employed. Since such models are not flawless, several data points can be erroneously classified. As a result, an estimation of accuracy is mandatory usually via a confusion matrix (a tabular summary presenting the model’s value). In the current study each fold, confusion matrix and statistical parameters (Equations (3)–(7)) on the independent test dataset were applied for analyzing the efficiency of the superlative VGG16-based model. The average of the whole five folds was used for evaluation of the total efficiency of the CNN model.

**Figure 4:**
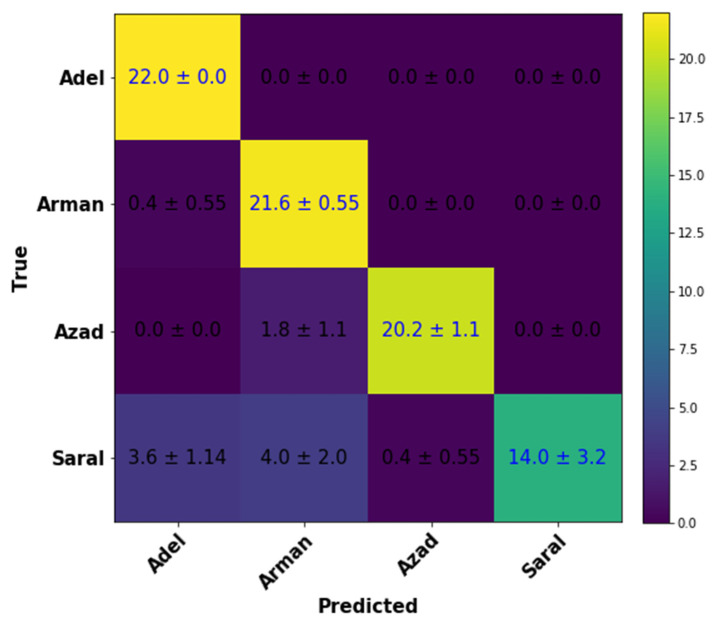
Average confusion matrix evaluated from the proposed Convolutional Neural Network model with four various classes.

To further verify the model’s versatility in total four chickpea varieties were included (Adel, Arman, Azad, and Saral). Due to the comparable shape of diverse varieties of chickpea, it is somewhat hard to discriminate them with morphologically using just the naked eye. Based on the constructed model, the identification accuracy levels were satisfactory. Figure 4 presents the average value of confusion matrices, denoting that chickpea variety identification and discrimination was correctly done and with high accuracy. The class of a single variety (Saral) included additional misclassified images, which were confused with images of other variety classes.

By classification of the test dataset into four classes, the average values of statistical parameters accuracy, precision, sensitivity, specificity, and AUC were 94.21, 90.49, 96.13, 88.41, and 92.27, respectively (Table 2). The obtained results of the developed model assessment in different folds show that the introduced model has the required performance for chickpea variety identification and discrimination.

### 3.2. Qualitative Analysis

The training process of the CNN models comprises two feed-forward and back-propagation steps. In back-propagation step, learning these models for optimization of their kernels (filters) is performed. Low- and high-level features are extracted by these optimized kernels. They also demonstrate the most significant regions for recognizing chickpea variety images for each class. Therefore, the accuracy of the models is specified by kernels, and visualization of kernels is essential for assessing the model performance.

**Figure 5:**
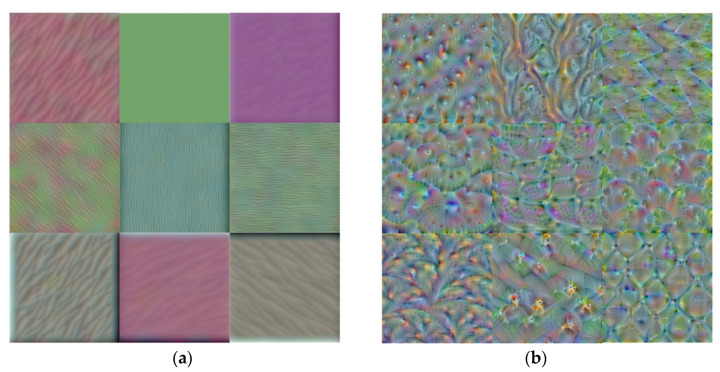
Visualizing nine filters of the first (**a**) and last (**b**) convolutional layers.

Extracted filters or features of diverse layers, by using the model, are applied to determine the ultimate accuracy of the modified model. Assessment of the visual patterns of the filters’ response is known as an appropriate method for estimation of the modified model performance in order to extract low- and high-level features. To achieve this goal, different filters’ extraction of diverse convolutional layers of the model was done. Particular filters were applied in specific filters of the first and last convolutional layers (Figure 5). The first layer filters encoded the colorful features and directional edges (Figure 5). Texture particular patterns of the images, which were constructed of colors’ and edges’ compositions, were encoded by the last convolutional layers’ filters (Figure 5). Therefore, the modified model according to the shape, color, and texture has simulated various effective filters.

The Gradient-weighted Class Activation Mapping (Grad-CAM) is an efficient method for debugging the prediction process. The Grad-CAM was utilized for visualization of the regions of random input images used for feature extraction, which was employed for prediction of the images’ class. By using this technique, a class activation heatmap of the input image was created and computed for all of its locations. The 2D grid class activation heatmap was used for specifying the significance grade and resemblance level of each image location according to the particular class.

**Figure 6:**
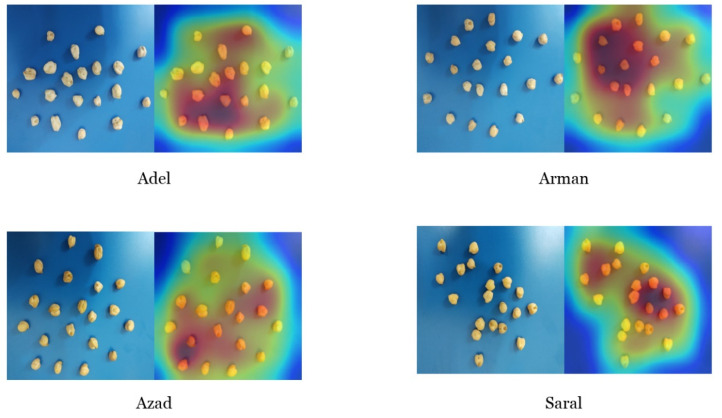
Gradient-weighted Class Activation Mapping visualization for each original image of every class.

To acquire the class activation heatmap, initially the feature map extraction of the final convolution layer for a specified image was done, and the gradient of the particular class was calculated by considering the feature map of the convolutional layer. Next, the neuron weights were computed by using these gradients. Eventually, the weight of any channel of the feature map was calculated by using these weights. The results of applying this technique on the selected random images of the four different chickpea varieties are displayed in Figure 6. The CNN model had the capability for accurate feature extraction of the right location (Figure 6). This accurate feature extraction of the appropriate location was feasible, even though the camera was placed distantly from the samples. This fact fortifies the ability and consistency of the optimized model.

**Figure 7:**
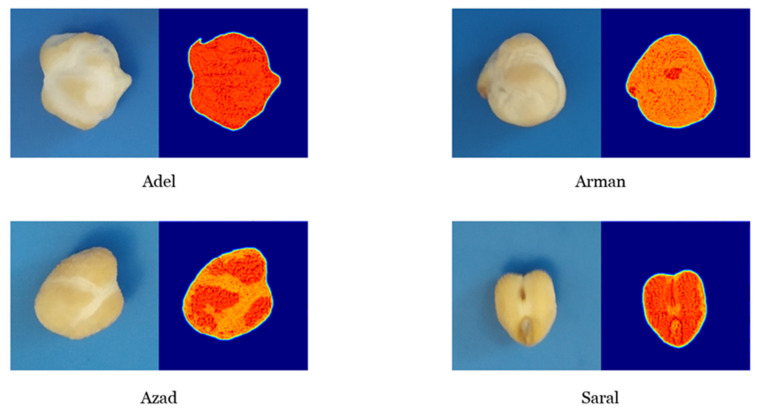
The class saliency map visualization of channel 46 from the first convolutional layer of the Convolutional Neural Network model.

In order to diagnose the features from each class of an image, which were extracted by the CNN model from any given sample, the content of a single sample was manually isolated. This manually cut sample was evaluated with the CNN model. Next, the class saliency map of these images was computed. The class saliency map consisted of 46 and 488 channels for the first and last convolutional layers, respectively (Figure 6 and Figure 7).

The class saliency map is used for pixel visualization of an image, which assists in the classification. Initially, the derivative of class score function was computed for an input image via backpropagation, having m rows and n columns. The First-order Taylor expansion adequately approximates the class score function as a linear function of the specified image. Next, the elements of the computed derivative were rearranged to obtain the saliency map. Every component of the saliency map in the RGB image is the uppermost value of the calculated derivative among the three-color channels.

**Figure 8:**
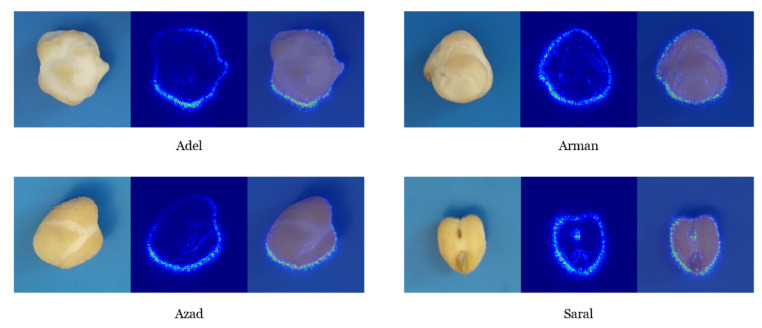
The class saliency map visualization of channel 488 from the last convolutional layer of the Convolutional Neural Network model.

Particular filters in the first layer, which were learned, represent the surface color (Figure 7). Several low-level features (e.g., colors, edges, and colored edges) can be extracted by these layers. The possibility of the filters’ focus on the sample color activated neurons on the sample surface. The entire boundary of the sample is denoted in Figure 8. The activated neurons on the sample outline serve as a set of gradient operators, which are employed for detection and extraction of the sample edge structure at diverse orientations. The extraction of more complex levels of features was done based on the deeper layer (i.e., last convolutional layer). A set of self-learned features from low- to high- levels was seen at the feature visualization and class saliency maps. Consequently, distinguishing the intricate visual features of the diverse chickpea varieties and recognizing them according to these features is conceivable by CNN models.

High-level understanding from digital images, and recognition of objects in particular, has been the epicenter of computer vision in recent years, and progress on that field has been exponential. Particularly, the Pascal Visual Object Classes (VOC) challenge [36], and lately the Large-Scale Visual Recognition Challenge (ILSVRC) [23] founded on the ImageNet dataset [37] are extensively employed as standards for several visualization-related enigmas in computer vision, and especially object sorting. In the past decade, a large, deep CNN reached a 16.4% error for imaging classification into thousands of probable classes [25]. In the succeeding years, several progresses in deep CNN depressed the error rate to 3.57% [26,28,38]. The prediction efficiency recorded in this study was at the highest end of the range, when considering previous reports assessing seed variety identification and discrimination by using other methods (67.5–91.3% accuracy of the classification results for test datasets; [39,40,41,42,43]. Even though training large neural networks can be proven a laborious process, nevertheless the accomplished models have the ability to swiftly recognize and categorize images, making them suitable for smartphones applications.

## 4. Conclusions

Rapid seed variety determination remains a difficult undertaking in agricultural sector. It often relies on human experts, and in this way is a subjective and error-prone process. The proposed Convolutional Neural Network employs a powerful deep learning model alternative in order to establish the deep relation among the input data and the target output. The competence of the model was correspondingly established by experimental results. In addition, the proposed vision-based model was proven very robust and flexible for seed variety identification, since two different types of cameras were used for imaging, while light and image acquisition (e.g., distance, angle, background) conditions were variable. Instead, feature engineering-based approaches require defined illumination and image acquisition conditions. Therefore, it was established that the required imaging data can be acquired by using easily accessible devices such as mobile phones, tablets, or action cameras (GoPro). In this way, there is a potential that a smartphone may evolve as a very flexible alternative and a seed variety identification platform, via developing a CNN model-based mobile app. Our results indicate that the proposed CNN algorithm is reliable and very efficient for straightforward seed variety identification and discrimination, thus showing a great potential for application in seed producers, processors, and distributors. This presents a great potential for extension into new applications using mobile phones to acquire and process information *in situ*. The proposed protocol consists of a smart, non-invasive, and reliable technique for online identification of chickpea seed varieties.

## Figures and Tables

**Table 1:** Comparison of the proposed Convolutional Neural Network performance in each fold.

Fold	Training		Validation		Testing	
	Accuracy	Loss	Accuracy	Loss	Accuracy	Loss
1	0.9234	0.2364	0.9062	0.2501	0.8977	0.3708
2	0.9133	0.2417	0.9062	0.2481	0.8636	0.4368
3	0.9294	0.2136	0.9375	0.1731	0.8636	0.3889
4	0.8909	0.3086	0.9667	0.1117	0.9318	0.3477
5	0.9087	0.2643	0.9500	0.1679	0.8636	0.4275
Average	0.9131 ± 0.01	0.2529 ± 0.03	0.9333 ± 0.02	0.1902 ± 0.05	0.8841 ± 0.03	0.3943 ± 0.03

**Table 2:** Average statistical parameters of proposed Convolutional Neural Network model.

Class	Accuracy (%)	Precision (%)	Sensitivity (%)	Specificity (%)	AUC (%)
Adel	95.46 ± 1.13	84.72 ± 3.26	100 ± 0.00	93.94 ± 1.51	96.97 ± 0.76
Arman	92.96 ± 2.03	79.06 ± 5.09	98.18 ± 2.49	91.21 ± 2.49	94.69 ± 1.85
Azad	97.5 ± 0.95	98.18 ± 2.49	91.82 ± 4.98	99.39 ± 0.83	95.61 ± 2.23
Saral	90.91 ± 3.68	100 ± 0.00	63.64 ± 14.72	100 ± 0.00	81.82 ± 7.36
Average per-class	94.21 ± 1.52	90.49 ± 2.24	88.41 ± 3.05	96.13 ± 1.01	92.27 ± 2.03

## Data Availability

All relevant data are included in the manuscript. Raw images are available on request from the corresponding author.

## Supplementary Materials

The following are available online at https://www.mdpi.com/article/10.3390/plants10071406/s1, Figure S1: Progression of mean accuracy and loss score through the period of 100 epochs across all experiments.

## Abbreviations

*AUC*	area under the curve
*CNN*	convolutional neural network
*FN*	false negatives
*FP*	false positives
Grad-CAM	Gradient-weighted Class Activation Mapping
ReLU	Rectified Linear Unit
RGB	red, green and blue
*TN*	True negatives
*TP*	True positives
