# Supplementary material for: Automated In Situ Seed Variety Identification via Deep Learning: A Case Study in Chickpea
Source: Plants (Basel). 2021 Jul 9;10(7):1406. doi: 10.3390/plants10071406 (PMC8309301; doi:10.3390/plants10071406)
Supplement: Supplementary file 1 [file plants-10-01406-s001.zip › plants-1271586-supplementary.pdf]

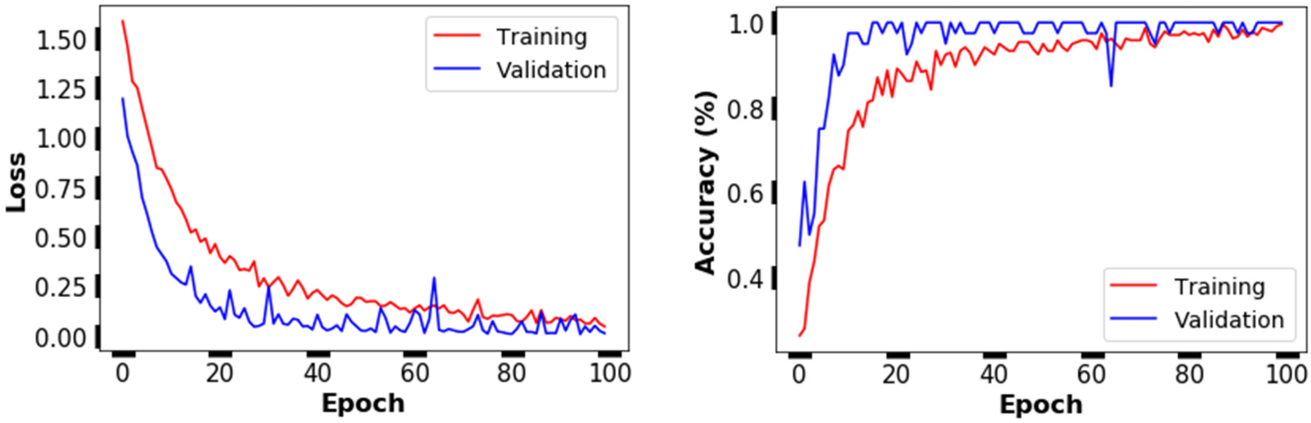

Supplementary figure S1: Progression of mean accuracy and loss score through the period of 100 epochs across all experiments.
